# Administration of GnRH at day 20 of AI and embryonic losses in synchronized crossbred buffalo

**DOI:** 10.3389/fvets.2023.1118865

**Published:** 2023-02-23

**Authors:** Adili Abulaiti, Zahid Naseer, Wenju Liu, Zulfiqar Ahmed, Mohamed Abdelrahman, Aftab Shaukat, Xunsheng Pang, Shujuan Wang

**Affiliations:** ^1^College of Animal Science, Anhui Science and Technology University, Chuzhou, Anhui, China; ^2^Anhui Province Key Laboratory of Animal Nutritional Regulation and Health, Chuzhou, Anhui, China; ^3^Faculty of Veterinary and Animal Sciences, Pir Mehr Ali Shah Arid Agriculture University, Rawalpindi, Pakistan; ^4^College of Life and Health Science, Anhui Science and Technology University, Chuzhou, Anhui, China; ^5^Key Laboratory of Swine Genetics and Breeding of Ministry of Agriculture and Rural Affairs, and Key Laboratory of Agricultural Animal Genetics, Breeding and Reproduction of Ministry of Education, Huazhong Agricultural University, Wuhan, China; ^6^Animal Production Department, Faculty of Agriculture, Assiut University, Asyut, Egypt; ^7^Key Laboratory of Animal Genetics, Breeding and Reproduction, Ministry of Education, College of Animal Science and Technology, Huazhong Agricultural University, Wuhan, China

**Keywords:** crossbred buffaloes, GnRH, progesterone (P4), embryonic mortality, estrus synchronization

## Abstract

**Introduction:**

Following the application of different artificial insemination and synchronization protocols, the synchronized buffaloes had a higher incidence of early embryonic or fetal death, thus impairing reproductive performance. Therefore, there is a need to devise a synchronizing program that can improve conception, allow early pregnancy diagnosis, reduce early embryonic losses, and provide an early clue for pregnancy diagnosis and establishment. The present study aimed (1) to determine the effect of administration of different GnRH doses at day 20 of artificial insemination (AI) on the reproductive performance of buffaloes and (2) to observe the influence of synchronization protocol, parity, age, milk production, and body condition score (BCS) on early embryonic loss and progesterone (P4) levels in crossbred buffaloes.

**Methods:**

Crossbred buffaloes (*n* = 101) were synchronized using the GPGMH protocol. At day 20 of AI, the synchronized buffaloes were randomly divided into four groups and administrated GnRH doses (0, 100, 150, and 200 μg). The buffaloes were monitored for ovarian dynamics, P4 level, non-return rate, embryonic/fetal losses, and pregnancy rates. The previously synchronized buffaloes were also classified for synchronization protocol (with or without GnRH), parity (nulli- or multiparous), milk production (high or low), BCS (low, medium, or good) or age (>3 or < 3 years) groups for observing the embryonic loss and P4 level variations.

**Results:**

The results indicated no difference (*P* > 0.05) in CL size, P4 level, pregnancy rate and embryo/fetal losses across the treatment groups at different observation periods. There was a high (*P* < 0.05) incidence of early embryonic mortality in aged, multiparous, low BCS and low milk-producing buffaloes treated without GnRH.

**Conclusion:**

The data suggest that GnRH 200 μg at day 20 of AI improves embryo survival and pregnancy maintenance in crossbred buffaloes.

## 1. Introduction

In buffalo production, embryonic mortality is a major cause of infertility (1–3). During the low breeding season, reproductive activity declines due to subtle estrus behavior, resulting in fewer cows cyclicity and conceiving (3). Therefore, buffalo breeding and calving in the Mediterranean region is timed when buffalo milk demand is greater (4). However, this reprogramming potentially affects the establishment of pregnancy, especially during the low breeding season. Even though various potential synchronization protocols have been used in buffaloes, conception per AI is low at around 30% in the low breeding season (5). Pregnancy rates increase to around 50% in animals synchronized during the breeding season (6). These findings highlight that buffalo can achieve higher pregnancy rates comparable to cattle after estrus synchronization (7).

Previously, it was observed that 45% of early embryonic loss occurred 26–40 days after AI in buffalo treated with the Ovsynch-TAI program (8). It is known that peak embryo losses occur during the maternal recognition period (12–18 days after fertilization; days 12 and 18 of AI) (9). The early embryonic loss period in cattle is up to 42 days after AI (10). The maintenance of CL is supported by the maternal recognition factor, which is also a critical time of 15–17 following AI and maintains the embryo's survival until the differentiation stage, at approximately 42 days of gestation (9). Various causes and mechanisms are involved in the loss of embryos in cows and buffaloes (11, 12). It is known that the success or failure of pregnancy depends on the circulating concentrations of P4 at maternal recognition and embryo implantation (13). Reduced secretion of P4 below a threshold is a cause of embryonic mortality in cattle (14) and buffaloes (12). In buffaloes, the plasma concentrations of P4 are typically lower and less reliable as an indicator of pregnancy during periods of increasing day length, which is associated with a higher incidence of early embryonic mortality (12). A higher concentration of P4 on day 10 after AI is observed in buffaloes with successful pregnancies than in those who lost the embryo at early stages. Meanwhile, the same phenomenon of P4 level is observed on day 20, after AI, in pregnant vs. non-pregnant buffaloes (3). Embryonic losses in cows and buffaloes have been reduced by applying different agents, GnRH, eCG, hCG, P4, and flunixin meglumine, in connection with synchronization protocols (15–18).

Mostly, the blood or milk P4 is quantified using radioimmunoassay (RIA) or enzyme-linked immunosorbent assays (ELISA) (19); however, its level is affected by abnormal conditions that can impact pregnancy results (20). Measuring circulating P4 concentrations provides a simple and economic tool to monitor embryo viability in buffalo. Moreover, the relationship between infectious agents and embryonic mortality is not accounted for by measuring the P4 concentrations. Therefore, the integration of ultrasonographic and hormonal analysis approaches could be useful for observing the structural variations in buffaloes with pregnancy or early embryonic losses (21). In this context, the present study aimed to investigate the effect of GnRH injection at day 20 of AI and possibly involved factors (synchronization, parity, age, milk production, and body condition score) on embryonic/fetal loss in Chinese crossbred buffaloes.

## 2. Material and methods

### 2.1. Animal care statement

The study was approved by the Animal Welfare and Ethical Committee of Huazhong Agriculture University, People's Republic of China (Approval ID: HZAUBU-2017-001). All experimental protocols were followed according to guidelines proposed by the Committee of Animal Research Institute, Huazhong Agricultural University, China.

### 2.2. Experimental site and climatic conditions

Crossbred buffaloes (Mediterranean × Murrah × Nili Ravi × Jianghan) were selected from a Hubei Jinniu Co., Ltd., Hubei province, China (latitude 30°32′N, longitude 111°51′E). The experiment was carried out from 1 October 2020 to 14 January 2021. The average ambient temperature varied between 5 and 19°C, with relative humidity ranging from 30 to 40% during summer.

### 2.3. Husbandry practices

A total of 101 (*n* = 101) adult (3–6 years age) multiparous (1–3 lactations) crossbred buffaloes with moderate body weight (621.12 ± 121.3 kg) and body condition score (2.5–3 points; 1–5 scale) (22), were randomly allocated into three treatment groups based on GnRH dosages. The selected buffaloes were clinically and physically healthy, normal history of reproductive soundness and showed regular estrus cycles, which were determined by tracking follicular development and ovulation through routine ultrasonography. All animals were housed in an open shed with a cemented rooftop and two sides fenced by galvanized wire mesh. The fans were installed for ventilation, and sprinklers for showering during peak heat periods; the grooming brush facilities were also available at the farm. A head-to-head stall-feeding system was in practice. Each animal was allocated an area of ~3.7 m^2^ and a manager of 0.6 by 0.9 m. Total mixed ration (TMR) was offered to buffaloes at a standard ratio (60:40) forage [corn silage (8%), peanut vine (16%), soybean (17%), rice straw (2%), corn (38%), soybean hulls (16%), flaxseed meal (6.0%), cottonseed cake (6%), cornmeal (17.5%), vinasse (10%), Sodium bicarbonate (0.5%) and mineral-vitamin premix (6%)]. Animals had free access to fresh, clean water.

### 2.4. Modified ovsynch estrus synchronization protocol and pregnancy diagnosis

**Figure 1:**
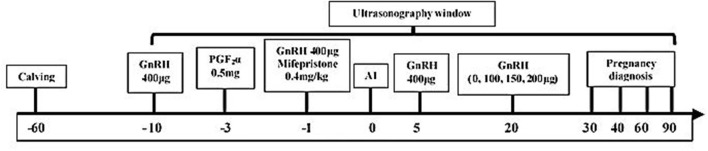
Layout describing the schedule of synchronization protocol, ultrasound monitorings, AI, post-AI hormonal treatments, and pregnancy diagnosis in buffaloes.

All animals (*n* = 101) were treated with a modified Ovsynch program [1st GnRH, (400 μg, intramuscular (IM); Ningbo Sansheng Pharmaceutical (NSP), China) at day −10, PGF_2_α (0.5 mg, IM; NSP) on the −3 day, 2nd GnRH injection (200 μg) and mifepristone (0.4 mg/kg BW, IM, Hubei Yun Cheng Sai Technology, China) on day −1, AI after 24 h of the 2nd GnRH using frozen-thawed semen and 3rd GnRH on the day 5th day of AI; Figure 1]. Ultrasonography (Desktop B-type veterinary ultrasound scanner, WED-9618-v, LV2-3/6.5 MHz rectal probe; Shenzhen Well D Medical Electronics, Guangdong, China) was used to monitor follicle development (from the day before PGF_2_α treatment until 72 h of the 2nd injection of GnRH), CL dynamics (at day 20 and 21 of AI). Prior to AI, the buffaloes were also monitored for estrus signs (vaginal mucous discharge, bellowing, milling, swollen vagina, and head butting) twice daily (06:00 and 18:00). Buffaloes were also observed for estrus signs between days 18 to 24 of AI to record the non-return rate in synchronized buffaloes.

The experimental buffaloes were further divided into four groups (GnRH-0; *n* = 26, GnRH-100; *n* = 22, GnRH-150; *n* = 25 and GnRH-200; *n* = 28) according to the dose of 4th GnRH injection (0, 100, 150, and 200 μg) at day 20 of AI. The buffaloes were monitored for pregnancy losses at day 30, 40, 60, and 90 days after AI using ultrasonography. Milk samples were collected at day 20 of AI for milk P4 level (Quanzhou Ruixin Biological Technology Co., LTD) to predict the pregnancy in synchronized buffaloes. In addition, blood samples were obtained from synchronized buffaloes at days 20, 40, and 60 to observe deviation in the P4 level of buffaloes with pregnancy or embryonic loss.

### 2.5. Determination of involved factors for embryonic loss and P4 level

The synchronized buffaloes were divided into the different categories [protocol (GnRH treatment 20 days after AI or untreated), parity (nulli- and multiparous), age (< 3 or >3 years), milk production (low vs. high), and pregnancy (pregnanct vs. non-pregnant)] to observe the variation in serum P4 levels and embryo losses at observed days.

### 2.6. Statistical analysis

Data were analyzed using statistical software (SPSS version 17.0.1 Chicago, IL, USA). One-way analysis of variance (ANOVA) was applied to compare the P4 level, CL size, and follicle size. Two-way ANOVA was used to analyze milk production and composition at different intervals among the groups. However, the chi-square test was applied to compare pregnancy, non-return rates, embryonic losses and pregnancy rates using the Prism-6 software package (GraphPad Software). An initial stepwise logistic regression model was constructed to determine the significant and non-significant association between independent (protocol, milk yield, age, parity, and BCS) and dependent variables (early embryonic losses and P4). Values of *P* < 0.05 were considered statistically significant.

## 3. Results

The effect of different doses of GnRH at day 20 of AI on estrus response, pregnancy rate, embryonic mortality, fetal losses and P4 level in buffaloes is presented in Table 1. It is observed that CL size and milk P4 were significantly different (*P* < 0.05) in GnRH-200 compared to the control group at day 20 of AI/day of the 4th injection of GnRH. There was no change in follicle size at day 20 of AI and non-return rates among the groups. Moreover, the pregnancy rates diagnosed at days 30, 40, 60, and 90 of AI were also the same across the treatment groups. Embryonic or fetal losses were also non-significant among the treatment groups observed at days 30, 40, 60, and 90 of AI. The serum P4 level was significantly higher (*P* < 0.05) in the GnRH-200 group than in other treatment groups on day 20 of observation but remained the same across the groups at different observational periods (days 40 and 60 of AI). The serum P4 level of pregnant buffaloes significantly improved (*P* < 0.05) in GnRH-200 treatment groups compared to the rest. Higher (*P* < 0.05) P4 levels were observed in non-pregnant buffaloes when treated with GnRH doses of 100, 150, and 200 μg compared to the control. However, the P4 level was not influenced by GnRH treatment in buffaloes with embryonic losses.

**Table 1:** Effect of different doses of GnRH at day 20 of AI on follicle or CL size, non-return rate, pregnancy rate, embryonic or fetal losses, and seum/milk P4 level in post-partum buffaloes.

**Variables**	**GnRH-0 (*n* = 26)**	**GnRH-100 (*n* = 22)**	**GnRH-150 (*n* = 25)**	**GnRH-200 (*n* = 28)**
Follicle size at day 20 of AI (mm)	8.2 ± 1.2	8.9 ± 1.3	8.7 ± 0.9	8.7 ± 0.9
CL size at day 20 of AI (mm)	9.5 ± 1.0^b^	9.8 ± 1.4^b^	9.6 ± 1.4^b^	10.9 ± 1.1^a^
Non-return rate (%)	12/26 (46.2)	11/22 (50.0)	14/25 (56.0)	19/28 (67.9)
Milk P4 at day 20 of AI (ng/ml)	2.91 ± 0.106^b^	3.89 ± 1.152^ab^	4.82 ± 0.852^a^	6.27 ± 1.346^a^
**Pregnancy rate (%)**				
Day 30	09/26 (34.6)	08/22 (36.4)	11/25 (44.0)	14/28 (50.0)
Day 40	08/26 (30.8)	07/22 (31.8)	10/25 (40.0)	13/28 (46.4)
Day 60	07/26 (26.9)	06/22 (27.3)	08/25 (32.0)	12/28 (42.9)
Day 90	05/26 (19.2)	05/22 (22.7)	08/25 (32.0)	12/28 (42.9)
**Embryo or fetal loss (%)**				
Day 30	03/12 (25.0)	03/11 (27.3)	03/14 (21.4)	05/19 (26.3)
Day 40	01/9 (11.1)	01/8 (12.5)	01/11 (9.1)	01/14 (7.14)
Day 60	02/8 (25.0)	01/7 (14.3)	02/9 (22.2)	01/13 (7.7)
Day 90	02/7 (28.5)	01/6 (16.6)	0/7 (0)	0/12 (0)
**Serum P4 level in buffaloes (ng/ml)**				
Day 20	12.5 ± 2.5^b^	12.2 ± 0.5^b^	12.1 ± 0.3^b^	14.5 ± 2.5^a^
Day 40	11.4 ± 9.0	11.3 ± 5.9	9.3 ± 5.1	11.2 ± 3.3
Day 60	8.9 ± 3.2	9.9 ± 5.3	8.9 ± 6.2	10.6 ± 3.8
Pregnant category	12.5 ± 2.5^b^	12.2 ± 0.5^b^	12.1 ± 0.3^b^	14.5 ± 2.5^a^
Non-pregnant category	3.7 ± 0.1^b^	5.2 ± 0.4^a^	5.1 ± 0.2^a^	5.4 ± 0.1^a^
Category with early embryonic loss	8.1 ± 2.1	8.5 ± 2.4	8.7 ± 1.5	9.8 ± 1.8

Different superscripts (a, b) showing the significance level (P < 0.05; P < 0.001) between the groups.

The influence of synchronization protocol, parity, body condition score, age, and milk production on embryonic losses in buffaloes is shown in Table 2. There was a significant effect (*P* < 0.05) of GnRH inclusion on embryonic survival between days 20 and 40 of AI compared to 40 days onwards. There was a tendency of overall high embryonic losses without GnRH treatment. Parity did not influence embryonic losses; however, higher (*P* < 0.05) embryonic mortalities were observed in nulliparous buffaloes. Low BCS buffaloes experienced overall higher (*P* < 0.05) embryonic losses compared to medium or good BCS animals. Low milk-producing buffaloes lost more conceptions (*P* < 0.05) at the embryonic stage compared to high milk-producers buffaloes. In addition, the older buffalo had higher pregnancy rates.

**Table 2:** Variation of pregnancy losses in buffaloes in response to synchronization protocol, parity, body condition score, age, and milk production.

**Factor**		**Embryo or fetal loss (%)**			

		**Day 20**	**Day 40**	**Day 60**	**Overall**
Synchronization protocol	With GnRH	8/75 (10.7)	3/67 (4.5)	4/64 (6.3)	15/75 (20.0)
	Without GnRH	3/26 (11.5)	4/23 (17.4)	3/20 (15.0)	10/26 (38.4)
	*P*-value	0.9021	0.0489	0.2830	0.0602
Parity	Nulliparous	6/42 (14.3)	4/36 (11.1)	5/32 (15.6)	15/42 (35.7)
	Multiparous	5/59 (8.5)	3/54 (5.6)	2/51 (3.9)	10/59 (16.9)
	*P*-value	0.3555	0.3866	0.0968	0.0313
Body condition score (BCS)	Low BCS	5/22 (22.7)	3/17 (13.6)	3/14 (13.6)	11/22 (50.0)
	Medium BCS	2/43 (4.7)	2/41 (4.7)	1/40 (2.3)	5/43 (11.6)
	Good BCS	4/36 (11.1)	2/32 (5.6)	3/29 (8.3)	9/36 (25.0)
	*P*-value	0.0261	0.1983	0.0726	0.0007
Milk production	High	4/63 (6.3)	2/61 (3.2)	3/58 (4.8)	7/63 (11.1)
	Low	7/38 (18.4)	5/31 (13.2)	4/27 (10.5)	16/38 (42.1)
	*P*-value	0.0492	0.0457	0.2692	0.0003
Age	< 3 years	7/42 (16.7)	4/35 (9.5)	5/31 (11.9)	16/42 (38.1)
	>3years	4/59 (6.8)	3/55 (5.1)	2/52 (3.4)	9/59 (15.2)
	*P*-value	0.1159	0.3866	0.0968	0.0088

The influence of synchronization protocol, parity, body condition score, age, milk production, and pregnancy status on changes in P4 levels in buffaloes is shown in Table 3. The synchronization protocol type did not affect the P4 level on days 20 and 40 of AI, but the P4 level increased on day 60 of AI when GnRH was included in the protocol. However, there was no effect of parity on the P4 level at days 20 and 40 of AI but an increased (*P* < 0.05) P4 level was observed in multiparous buffaloes. There was a significant influence (*P* < 0.05) of good BCS and pregnancy on the P4 level at each observational point. In contrast, milk production and age did not influence the P4 level at any point of observation.

**Table 3:** Variation of P4 levels in buffaloes in response to synchronization protocol, parity, body condition score, age, milk production, and pregnancy status.

**Factors**		**Serum P4 (ng/ml)**			

		**Day 20**	**Day 40**	**Day 60**	**Overall**
Synchronization protocol	With GnRH	11.2 ± 1.5	11.0 ± 1.2	11.3 ± 1.6	11.2 ± 1.4
	Without GnRH	11.5 ± 2.5	11.4 ± 9.0	8.9 ± 3.2	10.6 ± 4.9
	*P*-value	0.7198	0.5978	0.0189	0.2934
Parity	Nulliparous	12.7 ± 2.3	11.8 ± 4.5	9.8 ± 1.8	11.4 ± 2.9
	Multiparous	14.5 ± 2.5	11.2 ± 3.3	10.6 ± 3.8	12.1 ± 2.9
	*P*-value	0.7040	0.4704	0.0448	0.5105
Body condition score (BCS)	Low BCS	12.1 ± 0.3	9.3 ± 5.1	8.9 ± 6.2	10.1 ± 3.9
	Medium BCS	12.2 ± 0.5	11.3 ± 5.9	9.9 ± 5.3	11.1 ± 3.9
	Good BCS	14.5 ± 2.5	11.2 ± 3.3	10.6 ± 3.8	12.1 ± 3.2
	*P*-value	< 0.0001	0.0005	< 0.0013	< 0.0001
Milk production	High	12.2 ± 0.5	11.3 ± 5.9	9.9 ± 5.3	11.1 ± 3.9
	Low	11.1 ± 3.8	10.8 ± 4.8	8.5 ± 2.4	10.1 ± 3.7
	*P*-value	0.3214	0.4038	0.1506	0.2403
Age	< 3 years	11.7 ± 3.2	10.2 ± 5.0	9.1 ± 2.1	10.3 ± 3.4
	>3years	12.5 ± 2.5	11.4 ± 9.0	10.9 ± 3.2	11.6 ± 4.9
	*P*-value	0.6908	0.1506	0.3111	0.4208
Status (%)	Pregnant	14.5 ± 2.5	11.2 ± 3.3	10.6 ± 3.8	12.1 ± 3.2
	Non-pregnant	4.1 ± 0.2	3.9 ± 0.3	3.7 ± 0.1	3.9 ± 0.2
	*P*-value	< 0.0001	< 0.0001	< 0.0001	< 0.0001

## 4. Discussion

The present study showed that administering different doses of GnRH (100, 150, and 200 μg) on 20 after AI may be a good strategy to enhance embryonic survival 20 to 40 after fertilization by increasing the P4 levels in crossbred buffalo. Moreover, synchronization protocol, parity, BCS, milk production level, and age influence serum P4 levels or embryonic losses in buffaloes.

Here, CL size, milk or serum P4 level at day 20 of AI indicates that buffaloes treated with 200 μg larger CL have more serum P4 levels than other buffaloes. These variables provide the clue of the healthy ovulatory follicle and subsequent transformation to CL, particularly in the GnRH-200 synchronized group. The buffalo groups treated with different doses of GnRH had similar non-return rates with overall, 25% of embryonic losses between days 20 to 30 of AI, which is a critical period of embryonic attachment. These results were comparatively lesser than those reported earlier in buffaloes treated with hCG, GnRH or P4 at 23 or 25 of AI (3, 8, 15). In contrast, Arshad et al. (23) reported that using GnRH at 23 of AI significantly improved embryonic survival in buffaloes. The increasing dose level of GnRH was not useful for promoting embryonic survival and P4 level; however, irrespective of dose level, improved P4 level and subsequent embryo survival were noted in buffaloes. This observation indicates the useful application of GnRH on day 20 of AI in buffaloes. However, there is a dearth of information regarding the exact cause (infectious or non-infectious) of pregnancy loss in the present study. Besides, the pregnancy wastage, day 40 onward to 90 of AI, was similar across the groups, but there is a lesser trend of pregnancy losses in buffaloes treated with 200 μg GnRH dose that coincided with P4 levels as well. The effect also depicts the beneficial influence of GnRH dose in treated buffaloes. The P4 level concerning pregnancy status or embryonic loss revealed that administration of 200 μg GnRH is the effective dose to maintain high P4 in pregnant and non-pregnant animals and even in animals with embryonic loss at different observational points.

While considering the effect of various external factors on P4 level and embryonic loss, it is observed that synchronization protocol affected embryonic loss and P4 level between days 40 to 60 of AI. This observation is also in consonance with claims made earlier in cows (24, 25). Previously there were comparisons of different synchronization protocols between the groups, but we adopted the same basic protocol across the groups with modification GnRH administration. In the present study, improvements might be connected to the potency of GnRH injections. The prominent effect of parity on P4 level and pregnancy wastage was observed in the late embryonic stages. Opposite to this result is a report by Pérez-Mora et al. (24) which notified that multiparous showed higher embryonic losses than the nulliparous. Higher pregnancy wastage in multiparous could be linked to high production stress during early location periods. In the current scenario, higher pregnancy wastage in nulliparous buffaloes might be due to the selection of low animals with compromised body conditions. The effect of parity on the P4 level was revealed earlier by Ramadan et al. (26), illustrating the no prominent effect on the P4 level. Therefore, selecting female animals of any parity and good management conditions should be considered for better pregnancy rates.

We observed the significant differences for P4 and embryonic losses concerning low, medium, and high BCS of synchronized buffaloes. Previously, similar to this finding is a report by Lopez-Gatius et al. (27) which explained that the risk of late embryonic mortality is multiplied by 2.4 for each unit of body condition lost in lactating cows. Carvalho et al. (28) described that lower BCS near the first AI is associated with decreased fertility and P4 level in cows. Additionally, it is also observed that pregnancy rates increased good BCS at calving and AI in cows (29–31) and buffaloes (2, 32). The age of animals influenced the early embryonic loss in aged buffaloes but not the P4 level, whereas numerically low levels of P4 were recorded. In contrast, no effect of age was documented by earlier studies (33). Previously, Fernandez-Novo et al. (25) also observed the farm effect on pregnancy loss in dairy cows, whereas we did not record this effect because we performed the study on the same farm. To what extent of embryonic losses still causes serious economic losses to producers due to late rebreeding of female animals or increased culling rates if seasonal breeding patterns exist, like buffalo herds (34)?

## 5. Conclusion

In conclusion, administering 200 μg GnRH at day 20 day of AI promotes peripheral P4 concentration, in turn, maintains embryo development in crossbred buffaloes. In addition, the choice of synchronization protocol, parity, BCS and age are key influencing factors for early embryonic mortality and P4 levels in buffaloes.

## Data availability statement

The original contributions presented in the study are included in the article/supplementary material, further inquiries can be directed to the corresponding authors.

## Ethics statement

The animal study was reviewed and approved by the study was approved by the Animal Welfare and Ethical Committee of Huazhong Agriculture University, People's Republic of China (Approval ID: HZAUBU-2017-001). All experimental protocols were followed according to guidelines proposed by the Committee of Animal Research Institute, Huazhong Agricultural University, China. Written informed consent was obtained from the owners for the participation of their animals in this study.

## Author contributions

SW, XP, WL, MA, AS, and AA designed and conducted the experiment. AA and WL performed the experiment and collected the data. AA, ZN, and SW analyzed the data and wrote and revised the manuscript. All authors have reviewed and agreed to the published version of the manuscript.
